# Comparative Evaluation of Hanging Objects as Environmental Enrichment Tools on Broiler Behavior, Welfare, Growth, Serum Chemistry, and Meat Quality Traits

**DOI:** 10.3390/vetsci13040321

**Published:** 2026-03-26

**Authors:** Taslim Amin, Sobia Alyas, Muhammad Usman, Muhammad Abbas Khan, Tarek Amin Ebeid, Muhammad Waqas, Muhammad Muneeb, Mudassir Ahmad, Ali R. Al Sulaiman, Sohail Ahmad, Ala E. Abudabos

**Affiliations:** 1 Institute of Molecular Biology and Biotechnology, The University of Lahore, Lahore 54600, Pakistan; 2 Department of Poultry Production, Faculty of Animal Production and Technology, University of Veterinary and Animal Sciences, Lahore 54000, Pakistan; 3 Department of Animal and Poultry Production, College of Agriculture and Food, Qassim University, Buraydah 52555, Saudi Arabia; 4 Department of Animal Nutrition, Faculty of Animal Production and Technology, University of Veterinary and Animal Sciences, Lahore 54000, Pakistan; 5 Poultry Research Institute, Rawalpindi 46000, Pakistan; 6 School of Science and Technology, Nottingham Trent University, Nottingham NG1 5LT, UK; 7 Environmental Protection Technologies Institute, Sustainability and Environment Sector, King Abdulaziz City for Science and Technology, P.O. Box 6086, Riyadh 11442, Saudi Arabia; 8 Department of Food and Animal Sciences, College of Agriculture, Tennessee State University, Nashville, TN 37209, USA

**Keywords:** poultry, environmental enrichment, growth performance, blood biochemistry, meat quality, broiler welfare

## Abstract

This study evaluated the effects of different environmental enrichment tools on broiler chickens from 1 to 21 days of age. A total of 120,000 one-day-old broiler chicks were randomly assigned to five treatments, each consisting of four replicates with 6000 birds per replicate. Replicates were housed in pens measuring approximately 362 m^2^. The treatments included: T1, green balls (approximately 40 balls per pen); T2, hanging toys (8–10 toys per pen); T3, hanging strings (8–10 string bundles per pen); T4, rotational use of green balls, hanging toys, and strings at equivalent densities; and T5, a control group without enrichment. Data were collected on growth performance, foot health, behavioral activity, serum biochemical parameters, and carcass and meat quality traits. Birds provided with hanging enrichment tools showed significantly improved growth performance (*p* ≤ 0.05) compared with the control group. Among enriched treatments, T2 yielded the highest body weight and weight gain, as well as the lowest feed conversion ratio. Enrichment treatments also resulted in significant improvements (*p* ≤ 0.05) in carcass characteristics and selected meat quality parameters, including lightness (L*) and pH stability. Behavioral observations indicated substantially higher activity levels in enriched groups relative to the control. Toe damage scores differed significantly among treatments (*p* = 0.004), with the lowest scores observed in T1 and the highest in T4; however, no significant differences were detected in footpad dermatitis or hock burn scores (*p* > 0.05). In conclusion, hanging environmental enrichment tools, particularly hanging toys, can effectively enhance growth performance, behavioral activity, and selected carcass and meat quality traits in broiler production systems, while their effects on footpad health appear limited under the conditions of this study.

## Simple Summary

This study assessed the effects of simple environmental enrichment strategies on broiler chicken welfare, growth performance, and meat quality. Hanging enrichment devices, including green balls, plastic toys, and strings, were introduced to a large population of broilers to promote pecking and exploratory behaviors. Provision of environmental enrichment increased bird activity and resulted in differences in foot and toe conditions among treatments, although no significant differences were observed in the incidence of footpad dermatitis or hock burn. Broilers with access to enrichment demonstrated superior growth performance, characterized by higher body weight and improved feed conversion ratio compared with unenriched controls. Meat quality parameters were also positively affected, particularly meat color characteristics and pH stability. Overall, these findings suggest that simple, low-cost environmental enrichments, especially hanging toys, constitute an effective and practical approach to improving broiler productivity, behavioral activity, and selected welfare- and meat-quality traits.

## 1. Introduction

Over the past several decades, broiler production has emerged as the fastest-growing sector of global animal agriculture, surpassing 100 million tons of meat produced in 2020. This expansion has been driven by global population growth, shifts in dietary patterns, and increasing demand for animal-derived protein sources [1]. Compared with other livestock species, such as pigs and cattle, broiler chickens exhibit superior feed conversion efficiency and rapid growth rates, rendering broiler production highly economically competitive [2]. Despite these advantages, increasing consumer concern, particularly in industrialized countries, has focused on the animal welfare implications of intensive broiler production systems. In such systems, birds are commonly housed in large, high-density flocks that may exceed 20,000 individuals [3]. These environments typically lack structural enrichment and consist of flat flooring, often concrete, covered with litter materials such as peat, wood shavings, chopped straw, or similar substrates [4]. Feed and water are provided through automated delivery systems, and indoor environmental conditions, including temperature and ventilation, are carefully regulated to maximize growth performance [5]. Nevertheless, intensive broiler production is frequently associated with significant welfare and health challenges. Production-related disorders, including lameness, footpad dermatitis, and sudden cardiac death, commonly peak during the final week of the production cycle and are particularly prevalent under high stocking densities [6]. These conditions adversely affect productivity by increasing mortality rates, reducing growth performance, and compromising meat quality [7]. Rapid growth rates combined with low levels of physical activity further exacerbate the risk of lameness and footpad dermatitis, particularly in environments characterized by poor litter quality or excessive moisture [8]. Consequently, affected birds may experience pain and discomfort, leading to diminished welfare outcomes and substantial economic losses for producers.

Environmental enrichment, defined as modifications to the housing environment that enhance the biological functioning of captive animals, has been proposed as a practical strategy for improving animal welfare and production performance [9]. The primary objectives of environmental enrichment include promoting more effective use of the environment, encouraging species-specific behaviors, reducing or preventing the development of abnormal behaviors, and enhancing cognitive engagement and problem-solving capacity [10]. When applied within farm animal production systems, environmental enrichment requires additional considerations beyond those typical of laboratory or zoological settings. Enrichment strategies must not only support mental and physical health and facilitate the expression of natural behaviors, but also be feasible for implementation at a commercial scale and economically sustainable for producers [11,12]. In broiler chickens, environmental enrichment primarily targets low activity levels, as birds may spend up to 80% of their time inactive. By stimulating locomotion and promoting variation in resting postures, enrichment interventions can increase muscle use and may contribute to a reduction in leg-related disorders, including lameness and footpad dermatitis [13,14,15].

Increasing environmental complexity has been shown to elevate broiler activity levels, at least temporarily [16]. The higher activity attained under normal, stable housing settings following the introduction of enrichment materials is likely attributable to improved fulfillment of broilers’ behavioral needs. Additional benefits include improved health outcomes, reduced fear responses, and increased expression of positive, species-typical behaviors, such as wing flapping [17,18]. Consequently, substantial evidence indicates that environmental enrichment enhances physical health, improves affective states, and enables the expression of natural behaviors, thereby contributing to overall broiler welfare [19,20,21]. Against this background, the present study investigates the effects of novel hanging enrichment tools on broiler chickens. Specifically, the study aims to evaluate growth performance, behavior, foot condition, serum biochemical parameters, carcass characteristics, and meat quality traits in broilers reared with and without the provision of enrichment.

## 2. Materials and Methods

### 2.1. Experimental Design

The experiment was conducted in a commercial broiler house located in the Sahiwal region of Punjab, Pakistan. A total of 120,000 day-old straight-run broiler chicks (Ross 308) were used in a completely randomized design. Each treatment group comprised four replicate pens, with each pen housing 6000 birds, resulting in a total of 20 experimental pens (5 treatments × 4 replicates). The experimental treatments (Table 1) were as follows: T1, birds were provided with green balls suspended within the shed from day 1 to day 21; T2, birds were exposed to hanging toys positioned at the top of the aviary from day 1 to day 21; T3, birds were provided with hanging strings continuously from day 1 to day 21; T4, birds received a rotational composite environmental enrichment program consisting of green balls during days 1–7, hanging toys during days 8–14, and hanging strings during days 15–21; and T5, the control group, received no environmental enrichment throughout the experimental period.

**Figure 1:**
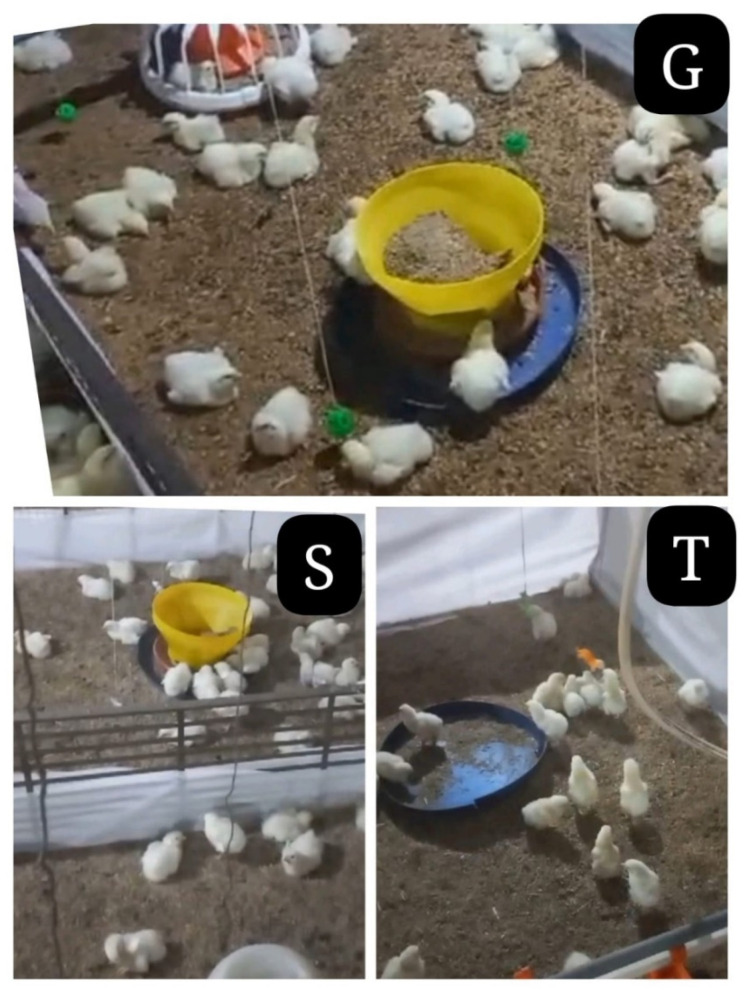
Environmental enrichment treatments applied in the study: G, green balls; S, hanging strings; T, hanging toys.

Each experimental replicate (pen) housed 6000 birds at a commercial stocking density of 0.06 m^2^ per bird, corresponding to a total pen area of 362 m^2^. To ensure separation between replicates, pens were divided using industrial white plastic sheeting. In addition, environmental enrichment items (Figure 1) were suspended from the shed ceiling and provided according to the assigned treatment. Specifically, T1 (green balls) comprised approximately 40 plastic pecking balls (6.8 cm diameter; green) per pen. These were distributed across 8–10 hanging points, evenly spaced throughout the pen (approximately one point every 457–610 cm). Multiple balls were attached to each hanging point to ensure adequate visibility and accessibility. By comparison, T2 (hanging toys) consisted of 8–10 identical plastic toys per pen, suspended from the ceiling and spaced approximately 457–610 cm apart. Similarly, T3 (hanging strings) included 8–10 bundles of white cotton string per pen, each bundle comprising 20 strands of 30 cm length, and suspended at a spacing comparable to that used for hanging toys. In contrast, T4 (rotational enrichment) followed a sequential enrichment protocol, with enrichment items provided at densities equivalent to those of the other treatments. For all enriched treatments, hanging objects were initially positioned at bird head height (approximately 20 cm above litter level at placement). Enrichment items were inspected daily for damage or wear, and their height was adjusted weekly in accordance with bird growth to maintain accessibility and promote interaction.

### 2.2. Bird’s Husbandry

**Figure 2:**
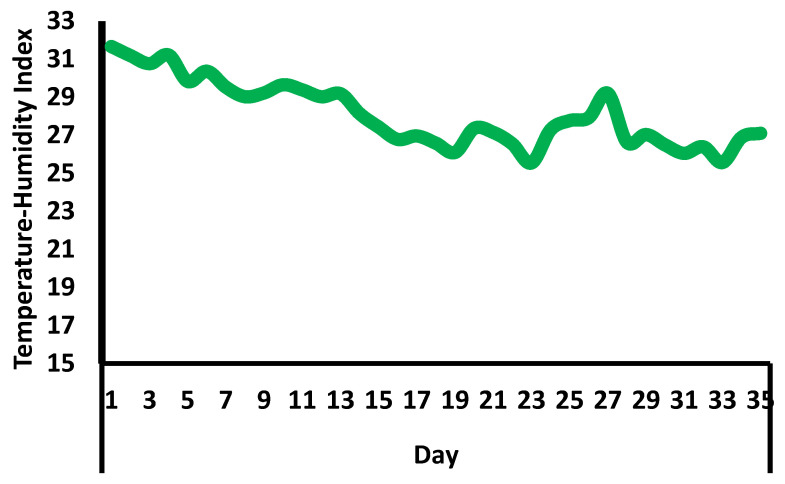
The trend of daily temperature-humidity index throughout the experimental period.

Experimental birds were housed in commercial broiler sheds equipped with modern environmental control systems, including automated ventilation, lighting, and temperature regulation. Ventilation was provided by exhaust fans and adjustable air inlets, while temperature was maintained using heaters and cooling systems. Ambient temperature and relative humidity were monitored daily to ensure that environmental conditions remained within recommended ranges for broiler chickens (Figure 2). A progressive lighting program was implemented. From day 1 to day 7, birds were maintained under a photoperiod of 23 h of light and 1 h of darkness. From day 8 to day 35, an intermittent lighting schedule of 18 h of light and 6 h of darkness was applied, delivered in repeated cycles of 3 h of light followed by 1 h of darkness. Chicks were provided with starter, grower, and finisher diets formulated to meet age-specific nutritional and physiological requirements. Feed and fresh drinking water were supplied ad libitum throughout the experimental period. Strict biosecurity measures, including routine disinfection and controlled access to the sheds, were enforced to minimize disease risk. Husbandry practices were standardized across all treatment groups to reduce potential confounding effects.

### 2.3. Parameters Evaluated

#### 2.3.1. Growth Performance

The effects of environmental enrichment on broiler productivity and overall production efficiency were evaluated over a 35-day experimental period using standard growth performance indicators. Feed intake (FI) was recorded daily and calculated as the difference between the total feed offered and the remaining feed. Body weight (BW) was measured weekly using a digital weighing scale (Wazan Tech, Gujranwala 52250, Pakistan; accuracy ± 0.5 g). Weekly body weight gain (WG) was determined as the difference between BW at the beginning and end of each week. Feed conversion ratio (FCR) was determined weekly as the ratio of FI to WG. Livability was assessed by recording mortality daily throughout the experimental period and expressed as the percentage of birds surviving relative to the number initially placed. The European production efficiency factor (EPEF) was calculated using the following equation:

EPEF = (Livability (%) × Live weight (kg))/(FCR × Market age (days)) × 100

#### 2.3.2. Behavioral Assessment

To evaluate the effects of hanging objects as environmental enrichment on the behavior of fast-growing broilers, behavioral observations were conducted using instantaneous scan sampling throughout the trial. Four focal birds were randomly selected from each replicate, individually marked with colored leg bands to facilitate identification and minimize observer bias, and then returned to their respective pens. These birds were monitored daily as focal individuals for detailed behavioral assessment.

Behavioral observations were conducted during four distinct time periods each day (08:00–09:00, 11:00–12:00, 14:00–15:00, and 17:00–18:00) to capture diurnal variation in activity patterns. Each observation session consisted of 5 min observation periods per focal bird, totaling eight observation periods per bird daily. All observations were performed by the same trained observer to ensure consistency. The observer stood approximately 2–3 m from the pen perimeter, remaining motionless for 2 min before recording to allow birds to acclimatize to their presence and resume normal behavior. To minimize disturbance, observations were conducted from outside the pen using visual contact through the industrial plastic sheeting dividers. The observer was blinded to treatment assignments during data collection and analysis to reduce potential bias. Behavioral data were collected according to a predefined ethogram (Table 2).

#### 2.3.3. Foot Condition

Broiler welfare was assessed on day 35 of the experiment. Ten birds were randomly selected from each replicate for evaluation. Welfare assessment focused on three-foot health indicators: toe damage, footpad dermatitis, and hock burn. Each parameter was scored using a binomial scale, where a score of 1 indicated the presence of the condition and a score of 2 indicated its absence. The scoring criteria were adapted from standard welfare assessment protocols (Welfare Quality^®^ Consortium) [22]. For toe damage, a score of 1 was assigned when visible lesions, swelling, or missing toes/portions of toes were observed, while a score of 2 indicated intact toes with no visible abnormalities. For footpad dermatitis, a score of 1 was given when lesions, discoloration, or scabs were present on the footpad, whereas a score of 2 indicated healthy, unblemished footpads. For hock burn, a score of 1 denoted the presence of discoloration, lesions, or scabs on the hock joint, and a score of 2 indicated clean, healthy hocks without lesions.

#### 2.3.4. Blood Biochemistry

On day 35, blood samples were collected from ten birds randomly selected from each replicate. Approximately 2 mL of blood was aseptically drawn from the jugular vein and transferred into EDTA tubes. For differential leukocyte counts, a drop of fresh blood was smeared onto a glass slide, air-dried, and stained using the Giemsa method. A total of one hundred leukocytes were counted across multiple fields per slide under a light microscope, and the heterophil-to-lymphocyte (H/L) ratio was calculated by dividing the number of heterophils by the number of lymphocytes.

For biochemical analysis, blood samples were collected from the wing vein, and serum was separated by centrifugation at 3000× *g* for 10 min at 4 °C. The harvested serum was stored at −20 °C until further analysis. Serum concentrations of total protein, albumin, glucose, and cholesterol were determined using commercially available assay kits (Randox Laboratories Ltd., Crumlin, UK) in accordance with the manufacturer’s instructions. The globulin concentration was calculated by subtracting albumin from total protein.

#### 2.3.5. Carcass Traits

On day 35 of the experiment, ten birds were randomly selected from each replicate and slaughtered in accordance with Halal guidelines (PS 3733:2016). Before slaughter, the individual’s live body weight was recorded using a high-precision electronic weighing scale to determine pre-slaughter weight. After slaughter, the shanks and visceral organs were removed, and the resulting dressed carcasses were weighed immediately. This weight was considered the edible carcass portion. Carcass yield was then calculated as the ratio of dressed carcass weight to pre-slaughter live weight using the following formula:

Carcass yield (%) = (Dressed weight (g))/(Pre-slaughter weight (g)) × 100

#### 2.3.6. Meat Quality

Meat quality parameters were evaluated on day 35 using breast muscle samples collected from ten birds per replicate. The assessed parameters included meat color attributes, lightness (L*), redness (a*), and yellowness (b*), as well as initial and ultimate pH. Muscle pH was measured postmortem and again at 24 h postmortem using a calibrated digital pH meter (WTW GmbH, Weilheim, Germany; model WTW 3210). For each sample, pH measurements were taken at three randomly selected locations, and the mean value was used for statistical analysis. Meat color was measured shortly after postmortem using a chroma meter (Konica Minolta Sensing Europe B.V., Warrington, UK, Konica Minolta Chroma Meter CR-41). Color measurements were obtained from three randomly selected positions on each sample, and the average values were calculated for subsequent statistical analysis.

### 2.4. Statistical Analysis

All collected data were initially examined for normality and homogeneity of variance. Normality was assessed using the Kolmogorov–Smirnov test, while homogeneity of variances was evaluated using Levene’s test. Data meeting parametric assumptions were subsequently analyzed using one-way analysis of variance with the General Linear Model (PROC GLM) procedure in SAS software (version 9.1, SAS Institute Inc., Cary, NC, USA). When significant treatment effects were detected, mean comparisons among treatment groups were performed using Tukey’s HSD test. Statistical significance was declared at *p* ≤ 0.05. Non-parametric one-way ANOVA was applied for the analysis of foot condition data, and associations between categorical variables were assessed using the chi-square (χ^2^) test. Because the enrichment sequence in T4 constituted a single rotational management strategy applied over the entire 21-day rearing period, T4 was analyzed as a single composite treatment, capturing the overall effect of the sequential enrichment program.

The following statistical model was used:

*Y_ij_* = *μ* + *τ_i_* + *ε_ij_*

where

*Y_ij_* = observation of the dependent variable in the *i*th treatment group;
*μ* = overall mean;
*τ_i_* = fixed effect of the *i*th treatment (*i* = 1, 2, 3, 4, 5);
*ε_ij_* = random residual error associated with the *j*th observation in the *i*th treatment group, assumed to be independent and normally distributed with mean 0 and variance $\sigma^{2}.$

## 3. Results

### 3.1. Growth Performance

During the first growth phase (days 1–21), FI was not significantly influenced by treatment (*p* = 0.247), with values ranging from 1162.71 g in T3 to 1179.27 g in T5. In contrast, BW, WG, FCR, and EPEF differed significantly among treatments (*p* < 0.0001). Broilers in T2 (hanging toys) and T4 (rotational enrichment) consistently exhibited superior performance compared with the other groups. These treatments yielded the highest BW and WG values, with BW of 812.2 g and WG of 772.4 g in T2, and BW of 806.6 g and WG of 766.4 g in T4. Correspondingly, EPEF values were significantly greater (*p* ≤ 0.05) in T2 (152.2) and T4 (151.3). Feed efficiency was also improved in these groups, as indicated by lower FCR values of 1.53 (T2) and 1.52 (T4). Conversely, the control group (T5; no environmental enrichment) demonstrated the poorest overall performance (*p* < 0.05), characterized by lower BW (690.7 g), WG (650.7 g), and EPEF (108.9), and the highest FCR (1.81). Treatments T1 (green balls) and T3 (hanging strings) showed intermediate performance, with BW and WG values significantly higher than those of T5 but lower than those observed in T2 and T4.

During the second growth phase (days 22–35), FI remained unaffected by treatment (*p* = 0.645), ranging from 1851.9 g (T4) to 1861.0 g (T3). However, significant differences persisted in BW, WG, FCR, and EPEF (*p* < 0.0001). The highest performance was again recorded in T2, which achieved a BW of 1933.9 g, WG of 1121.8 g, FCR of 1.66, and EPEF of 333.7. T4 ranked second, exhibiting slightly lower values than T2 but still significantly outperforming the remaining treatments. The control group continued to show the poorest performance, with the lightest BW (1750.8 g), WG (1060.06 g), EPEF (285.7), and the least efficient FCR (1.75). Across the entire 35-day experimental period, FI did not differ significantly among treatments (*p* = 0.437), ranging from 3019.8 g in T4 to 3035.2 g in T2. Nevertheless, WG, FCR, and EPEF differed markedly among treatments (*p* < 0.0001). T2 consistently demonstrated superior performance, recording the heaviest WG (1894.14 g), the highest EPEF (344.8), and the most efficient FCR (1.60). T4 ranked second and significantly outperformed T1, T3, and T5. As expected, the control group (T5) showed the lowest overall performance, with the lightest WG (1710.8 g), lowest EPEF (281.96), and poorest feed efficiency (FCR = 1.77). Treatments T1 and T3 exhibited intermediate performance levels (Table 3).

### 3.2. Behavioral Assessment

During the early growth phase (days 1–21), active behavior differed markedly among treatments, although differences among enriched groups were not always statistically significant. Birds in T4 (rotational enrichment) exhibited the highest proportion of active behavior (24.7%), followed by T1 (green balls; 24.1%) and T2 (hanging toys; 23.8%), while T3 (strings) showed slightly lower activity (22.7%). In contrast, the control group (T5) displayed the lowest activity level (6.9%). Correspondingly, inactivity was greatest in T5 (41.1%), significantly exceeding that of all enriched treatments, which showed comparable inactivity levels of approximately 26%. Eating behavior was most pronounced in T5 (25.0%), significantly higher than in all enriched groups, among which no significant differences were observed (e.g., T1 = 20.2%; T4 = 21.1%). Drinking behavior followed a similar pattern, with T5 recording the highest proportion of time spent drinking (21.8%), while enriched treatments did not differ significantly from one another, ranging from 19.5% (T1) to 20.2% (T4). Maintenance behavior varied among enrichment types, being highest in T3 (10.7%) and T2 (10.00%), whereas T4 (8.1%) and T5 (5.3%) showed significantly lower values, indicating differential effects of enrichment type on self-maintenance activities.

During the later growth phase (days 22–35), active behavior did not differ significantly among enriched treatments, with values of 23.8% in T1 and 23.3% in T2. However, T5 again exhibited significantly lower activity levels (6.4%) than all enriched groups. Inactivity was highest in T5 (42.2%), whereas enriched treatments showed lower and comparable inactivity levels, ranging from 21.5% (T2) to 22.30% (T3). Eating behavior remained most prominent in T5, while among enriched treatments, T4 recorded the highest eating activity, significantly exceeding that of T1, T2, and T3. Drinking behavior followed a similar trend, with T5 showing the highest proportion (23.8%), and enriched groups remaining statistically similar (19.27% in T4 to 19.88% in T3). Maintenance behavior was highest in T2 (14.5%) and T1 (14.1%), and lowest in T5 (5.1%). Across the entire experimental period (days 1–35), birds in T4 exhibited the highest overall activity level (24.18%), followed closely by T1 (24.00%), T2 (23.6%), and T3 (23.2%). The control group consistently showed the lowest activity (6.7%) and the highest inactivity (41.7%), significantly exceeding that observed in enriched treatments (23.8% to 24.2%). Eating behavior was most frequent in T5 (23.7%), whereas among enriched treatments, T4 recorded the highest proportion (21.5%), followed by T3 (20.8%), T2 (20.9%), and T1 (20.50%). Drinking behavior followed the same pattern, with T5 ranking highest (22.8%) and enriched treatments showing no significant differences (19.5% to 19.7%). Maintenance activities were highest in T2 (12.2%) and T3 (12.1%), intermediate in T4 (10.6%), and lowest in T5 (5.2%) (Table 4).

### 3.3. Foot Condition of Commercial Broiler

Toe damage scores differed significantly among treatments (*p* = 0.004). The highest mean score was observed in T4 (rotational enrichment using green balls, toys, and strings across growth phases; 1.89), followed by T2 (hanging toys for 21 days; 1.82). Intermediate scores were recorded in T3 (hanging strings for 21 days) and the control group, T5 (no enrichment), with values ranging from 1.69 to 1.75. The lowest toe damage score was observed in T1 (hanging green balls for 21 days; 1.64). In contrast, footpad dermatitis scores did not differ significantly among treatments (*p* = 0.051). Likewise, hock burn scores showed no significant treatment effects (*p* = 0.168), with mean values ranging from 1.76 to 1.90 (Table 5).

### 3.4. Blood Biochemical Profile

Broilers provided with environmental enrichment (T1: hanging green balls; T2: hanging toys; T3: hanging strings; T4: combined enrichment tools) exhibited significantly lower heterophil counts compared with the control group (T5; *p* < 0.0001). Heterophil values in enriched treatments ranged from 18.5 to 19.8, whereas the control group showed a markedly higher value (47.2). In contrast, lymphocyte counts were significantly greater in the control group (86.8) than in enriched treatments (*p* < 0.0001), which ranged from 70.1 (T4) to 76.2 (T1). Consistent with these results, the H/L ratio was significantly elevated in the control group (0.55) compared with all enriched treatments (*p* < 0.0001), which exhibited lower and comparable ratios (0.25–0.28).

Serum glucose concentrations followed a similar pattern, with significantly higher levels observed in T5 (204.6 mg/dL) compared with enriched treatments (*p* < 0.0001), which ranged from 183.9 mg/dL (T3) to 185.4 mg/dL (T2). Cholesterol concentrations were also highest in the control group (123.7 mg/dL) and significantly lower in enriched groups (*p* < 0.0001), with values ranging from 109.3 mg/dL (T4) to 111.3 mg/dL (T1). Furthermore, total serum protein levels were significantly greater (*p* < 0.0001) in the control group (5.95 g/dL) than in enriched treatments, which ranged from 4.68 g/dL (T2) to 4.73 g/dL (T3). Similarly, albumin and globulin concentrations, key indicators of protein metabolism and immune status, were significantly elevated in T5 (*p* < 0.0001). Albumin levels in the control group reached 3.14 g/dL, compared with 2.30 g/dL (T1) to 2.37 g/dL (T4) in enriched groups. Globulin concentrations were also highest in T5 (2.81 g/dL) and were reduced in enriched treatments, ranging from 2.32 g/dL (T2) to 2.38 g/dL (T1) (Table 6).

### 3.5. Carcass Traits

Pre-slaughter weight, dressed weight, carcass yield, liver weight, and heart weight of commercial broilers reared under different environmental enrichment treatments differed significantly among treatments, whereas gizzard weight was not affected. Pre-slaughter weight was significantly influenced by enrichment (*p* < 0.0001), with the greatest value observed in T2 (hanging toys; 1934.2 g), followed by T4 (rotational enrichment; 1896.2 g), T1 (green balls; 1853.68 g), and T3 (strings; 1833.4 g). The control group (T5) recorded the lightest pre-slaughter weight (1750.8 g), which was significantly lower than all enriched treatments. Dressed weight also differed significantly among treatments (*p* < 0.0001). Birds in T4 achieved the heaviest dressed weight (1268.2 g), followed by T2 (1240.0 g), T1 (1224.2 g), and T3 (1208.7 g). In contrast, the control group exhibited the lightest dressed weight (1090.1 g), significantly lower than all enriched groups. Carcass yield percentage was significantly higher in enriched treatments compared with the control (*p* = 0.028). The lowest yield was observed in the control group (62.3), whereas enriched treatments achieved higher values, with the highest carcass yield recorded in T4 (66.03%), followed by T1 (65.9%), T3 (65.6%), and T2 (64.1%). Differences among enriched treatments were not statistically significant (*p* > 0.05).

Liver weight was significantly affected by treatment (*p* < 0.0001), with the greatest value observed in T2 (46.2 g), followed by T4 (43.7 g), T1 (43.3 g), and T3 (38.9 g). The control group exhibited the lightest liver weight (35.0 g). A similar pattern was observed for heart weight, which was significantly greater in enriched treatments (*p* = 0.002). The heaviest heart weight was recorded in T2 (8.7 g), followed by T4 (8.0 g), T3 (7.9 g), and T1 (7.9 g), whereas the control group had the lowest value (6.8 g). In contrast, gizzard weight was not significantly influenced by environmental enrichment (*p* = 0.471), with values ranging from 21.1 g in T3 to 26.0 g in T1 (Table 7).

### 3.6. Meat Quality Traits

Commercial broilers reared under different environmental enrichment treatments exhibited significant differences in several meat quality attributes, including L*, initial pH, and ultimate pH, whereas a* and b* were not significantly affected. Lightness (L*) values differed significantly among treatments (*p* < 0.0001). All enriched groups showed significantly higher L* values compared with the control group. The control group (T5) exhibited the lowest L* value (50.8), whereas the highest L* value was observed in T3 (hanging strings; 55.5). Significant differences in L* were also detected among enriched treatments, particularly between T1 and T5. In contrast, a* and b* values did not differ significantly among treatments (*p* > 0.05). Redness values ranged from 13.1 in T3 to 14.3 in T4, whereas yellowness values varied from 16.02 in T3 to 18.5 in T2.

Initial pH was significantly higher in enriched groups compared with the control (*p* < 0.0001). Enriched treatments (T1–T4) exhibited similar initial pH values, ranging from 6.41 to 6.43, whereas the control group (T5) had a significantly lower initial pH (6.25). These results suggest improved pre-slaughter physiological stability in birds exposed to enrichment. Treatments also significantly influenced ultimate pH (*p* < 0.0001). The control group recorded the highest ultimate pH (5.72), potentially reflecting stress-related alterations in postmortem muscle metabolism. Among enriched treatments, T2 exhibited the highest ultimate pH (5.63), followed by T3 (5.60), T4 (5.59), and T1 (5.56), all of which were significantly lower than the control group (Table 8).

## 4. Discussion

Growth performance, as reflected by body weight and feed efficiency, was significantly enhanced in broilers provided with string enrichment compared with the control and other treatment groups. Body weight (BW) increased steadily throughout the production cycle, with string-enriched birds consistently exhibiting greater weight gains. In parallel, FCR was improved, indicating more efficient utilization of feed resources. These improvements may be attributed to increased physical activity and behavioral engagement stimulated by string enrichment, which may enhance metabolic efficiency and gastrointestinal function. Strings appear to serve as effective environmental enrichments by reducing boredom, encouraging natural pecking behavior, and mitigating stress. Reduced stress is closely associated with improved growth performance, as elevated corticosterone levels are known to suppress feed intake and impair nutrient absorption. Moreover, environmental enrichment has been linked to enhanced digestive enzyme activity, potentially further improving nutrient assimilation. Enrichment-induced sensory stimulation may also support neurocognitive development and reduce the occurrence of maladaptive behaviors such as feather pecking and cannibalism, both of which negatively affect feed intake and growth. In the absence of enrichment, control birds may allocate more time to non-productive activities or engage in feed-wasting behaviors; however, direct measurement of feed wastage was beyond the scope of the present study. The present findings are consistent with previous reports. Yenilmez et al. [23] demonstrated that hanging enrichment objects increased broiler activity and growth performance, while Riber et al. [24] reported improvements in feed efficiency and final body weight following enrichment provision. Similarly, Brantsæter et al. [25] showed that pecking-related enrichments enhanced growth rate and feed utilization. In contrast, Kemper and Tetens [26] observed no significant growth benefits, potentially due to differences in enrichment type, bird genotype, or management conditions. Tahamtani et al. [27] further emphasized that enrichment effectiveness depends on its complexity and novelty, indicating that not all enrichment strategies uniformly enhance performance. Overall, these results support the conclusion that appropriately designed and implemented environmental enrichment can improve broiler growth performance, highlighting its potential value as a practical management strategy in commercial production systems.

Broilers provided with string enrichment exhibited significantly higher levels of exploratory and foraging-related behaviors, including pecking, scratching, and perching, compared with birds in the control group. In addition, enriched birds displayed reduced aggression and fewer abnormal repetitive behaviors, whereas control birds showed higher frequencies of feather pecking and pacing. Time devoted to active behaviors was consistently greater in enriched birds, while sedentary behaviors, such as prolonged sitting, were more prevalent among non-enriched birds. The provision of string enrichment appeared to effectively satisfy the birds’ intrinsic motivation to explore and peck by redirecting these behaviors toward an appropriate substrate. Chickens possess a strong innate drive for exploratory and pecking activities; in environments lacking sufficient stimulation, this motivation may be redirected toward conspecifics, leading to aggressive interactions and feather damage. The availability of string enrichment likely provided a suitable outlet for pecking behavior, thereby reducing frustration, stress, and the expression of maladaptive behaviors. Increased interaction with enrichment materials may also have enhanced cognitive stimulation, contributing to improved welfare outcomes. Moreover, higher levels of activity are widely recognized as indicators of good health and environmental comfort, suggesting that birds in enriched conditions experienced a more favorable living environment. These findings are consistent with previous research. Nicol et al. [28] reported that environmental enrichment reduces the incidence of abnormal behaviors and improves poultry welfare. Tahamtani et al. [27] demonstrated that increased environmental complexity promotes species-specific behaviors while reducing fearfulness and aggression in broilers. Similarly, Lambton et al. [29] observed lower rates of injurious pecking in enriched flocks. In contrast, Kristensen et al. [30] found that enrichment does not always result in sustained behavioral changes, potentially due to habituation or loss of novelty over time. This limitation may be addressed by periodically modifying the type or placement of enrichment materials to maintain birds’ interest and behavioral engagement.

Assessment of broiler foot condition revealed significant differences in toe damage among the enrichment treatments. Birds exposed to rotational enrichment involving green balls, toys, and strings (T4) exhibited the highest toe damage scores, whereas broilers provided with a single, consistent enrichment (green balls for 21 days; T1) showed the lowest scores. In contrast, footpad dermatitis and hock burn scores did not differ significantly among treatments, indicating that these conditions were largely unaffected by the type of enrichment applied. The increased toe damage observed in the T4 group may be associated with the frequent alteration of enrichment materials. One possible explanation is that the periodic introduction of novel objects could have temporarily disrupted behavioral patterns, potentially leading to more vigorous or inappropriate interactions with enrichment items during initial exposure periods. This interpretation remains hypothetical, as the present study did not directly measure behavioral responses immediately following enrichment changes. However, previous research has shown that sudden changes in environmental complexity can temporarily increase activity levels and exploratory behavior [31], which might elevate the risk of minor injuries if interactions become overly vigorous. Alternatively, birds in the rotational treatment may have directed pecking behavior toward novel enrichment objects with greater intensity compared with birds habituated to a single enrichment type. These findings highlight that not all enrichment strategies produce uniformly positive welfare outcomes and that the timing and frequency of enrichment presentation may influence specific aspects of broiler welfare. Further research examining behavioral responses immediately following enrichment changes would help clarify the mechanisms underlying the observed toe damage differences. Conversely, the continuous provision of a single enrichment object, as implemented in T1, may have created a more predictable and stable environment, reducing stress-related behaviors and subsequent toe damage. These findings highlight that not all enrichment strategies produce uniformly positive welfare outcomes and that poorly designed or excessively complex enrichment programs may inadvertently compromise specific aspects of broiler welfare. The present findings are consistent with previous studies reporting variable effects of environmental enrichment on broiler welfare. Riber et al. [24] demonstrated that while enrichment can generally enhance welfare, rapidly changing or overly complex stimuli may induce stress and increase undesirable behaviors. Likewise, Yang et al. [32] reported no significant effect of enrichment on hock burn prevalence, supporting the present observation that hock burn may be relatively unresponsive to enrichment interventions. On the contrary, Schrader and Malchow [33] found that consistent enrichment provisions, such as perches or platforms, effectively reduced footpad dermatitis, whereas other types of enrichment showed limited efficacy. Collectively, these studies emphasize the importance of carefully designing and implementing enrichment strategies to optimize welfare outcomes in broiler production systems.

Significant differences were observed among treatments, particularly between enriched environments and the control group, indicating that environmental enrichment plays an important role in alleviating stress and improving the physiological status of broiler chickens. Birds provided with string-based enrichment (T3) exhibited the lowest concentrations of blood glucose, cholesterol, total protein, albumin, and globulin compared with all other treatments, including the non-enriched control group (T5). In addition, the H/L ratio, widely recognized as a reliable indicator of physiological stress, was markedly lower in the string-enriched group, further indicating reduced stress levels. It should be acknowledged, however, that blood biochemical parameters are influenced by multiple interacting factors, including nutritional status, metabolic rate, immune function, and circadian rhythms, in addition to stress responses [34]. Elevated blood glucose concentrations are commonly associated with stress-induced activation of glucocorticoids, which stimulate gluconeogenesis and glucose mobilization. The reduced biochemical values observed in string-enriched birds may therefore reflect a calmer physiological state, likely facilitated by continuous and appropriate environmental stimulation. Hanging strings may have promoted the expression of natural behaviors, such as pecking and exploration, thereby diverting attention from environmental stressors and attenuating activation of the hypothalamic–pituitary–adrenal axis, which plays a central role in regulating glucose and lipid metabolism. Lower albumin and globulin concentrations in enriched birds may indicate reduced metabolic demand and a more balanced immune status, free from chronic stress or inflammatory challenge. When considered alongside the reduced H/L ratio, these findings collectively suggest improved overall welfare in birds exposed to string enrichment. The present results are consistent with previous reports. Riber et al. [24] demonstrated that environmental enrichment in broiler systems improves welfare and reduces physiological stress, as reflected by favorable blood profiles. Similarly, Tahamtani et al. [35] reported that increased environmental complexity enhanced coping capacity and reduced stress indicators in broilers. Kang et al. [18] also observed improvements in metabolic and stress-related parameters when broilers had access to stimulating features such as pecking devices and platforms. However, not all studies have reported consistent benefits; Ventura et al. [36] found that certain enrichment strategies had minimal or no impact on stress responses or blood chemistry, suggesting that enrichment effectiveness depends on both enrichment type and sustained novelty. Collectively, these findings emphasize the importance of carefully selecting and managing enrichment strategies to ensure sustained engagement and optimal welfare outcomes in broiler production systems.

Environmental enrichment had a significant effect on pre-slaughter body weight, with the greatest value recorded in T2 (hanging toys for 21 days) and the lowest in the non-enriched control group (T5). These findings indicate that environmental enrichment plays a crucial role in promoting improved growth performance, likely through stress reduction and enhanced feeding motivation, which together support more efficient feed utilization. Among the enrichment strategies evaluated, hanging toys (T2) produced the most pronounced increase in pre-slaughter weight, suggesting that this form of enrichment was particularly effective in stimulating growth-related responses. Environmental enrichment also positively influenced dressing percentage and carcass yield. The greatest carcass yield was observed in T4, which received a combination of enrichment items, whereas the lowest yield occurred in the control group. Improved carcass yield in enriched birds may reflect enhanced nutrient absorption and metabolic efficiency associated with reduced physiological stress. Lower stress levels are known to improve endocrine balance, favor protein accretion, and limit catabolic processes, thereby supporting muscle development. Liver and heart weights were significantly greater in enriched treatments, with T2 exhibiting the greatest value, while the control group consistently recorded the lightest organ weights. Increased liver weight in enriched birds may be associated with reduced stress and increased feeding activity, resulting in greater metabolic engagement. Similarly, higher heart weights may indicate improved cardiovascular development, which can support enhanced growth and overall physiological resilience. In contrast, gizzard weight did not differ significantly among treatments, suggesting that environmental enrichment has a limited influence on gizzard development, which is more strongly determined by genetic background and dietary structure. Collectively, T2 (hanging toys for 21 days) and T4 (combined enrichment strategy for up to 21 days) produced the most favorable outcomes in terms of growth performance, carcass characteristics, and organ development, highlighting their potential applicability in commercial broiler production systems. These findings are consistent with previous reports. Ventura et al. [36] demonstrated that enriched environments improve carcass characteristics through enhanced muscle growth, while Bizeray et al. [37] reported increased breast muscle yield and reduced fat deposition in broilers reared in enriched pens. In contrast, Kells et al. [38] observed minimal differences in carcass traits between enriched and non-enriched systems, suggesting that the effectiveness of enrichment may depend on factors such as enrichment type, duration of exposure, and genetic line.

The present study demonstrated that environmental enrichment significantly influenced several meat quality traits in commercial broilers. Lightness (L*) values were highest in birds provided with hanging strings, whereas a* and b* did not differ significantly among treatments. The increased L* values observed in enriched groups suggest reduced pre-slaughter stress, which may have favorably influenced muscle pigmentation and water-holding capacity. Reduced stress is known to promote physiological stability, thereby positively affecting postmortem muscle characteristics. In contrast, the absence of significant differences in redness and yellowness indicates that these color parameters are likely more strongly influenced by genetic background, dietary composition, and processing conditions than by environmental enrichment. Initial muscle pH values were significantly higher in all enriched treatments, while ultimate pH values were lower in enriched groups compared with the control group. Higher initial pH values in enriched birds suggest improved preservation of muscle glycogen reserves as a result of reduced pre-slaughter stress, thereby limiting excessive lactic acid accumulation immediately following slaughter. The relationship between stress and ultimate pH is complex and depends on the timing and nature of stress exposure. Acute stress immediately before slaughter can deplete muscle glycogen reserves, resulting in reduced lactic acid production postmortem and consequently higher ultimate pH (dark, firm, dry meat) [39]. Conversely, chronic stress may lead to heightened glycogenolysis and greater lactic acid accumulation, producing lower ultimate pH [40]. The lower ultimate pH values observed in enriched treatments in the present study (5.56–5.63) compared with the control (5.72) may reflect more regulated postmortem metabolism and reduced chronic stress exposure throughout the rearing period, contributing to improved meat tenderness, enhanced water-holding capacity, and a reduced risk of pale, soft, exudative meat [41]. These findings are consistent with previous reports demonstrating that stress reduction through environmental enrichment positively influences muscle pH dynamics [24]. Chen et al. [42] observed higher initial pH values in broilers housed in enriched environments, indicating improved muscle energy preservation before slaughter. Conversely, the lack of changes in redness and yellowness is consistent with the findings of Simsek et al. [43], who emphasized that these traits are predominantly influenced by genetic and dietary factors rather than environmental conditions. Collectively, these results support the conclusion that environmental enrichment enhances broiler meat quality primarily by reducing stress and improving physiological status, although not all color parameters are equally affected.

## 5. Conclusions

This study demonstrates that environmental enrichment significantly influences broiler performance, behavior, welfare, and meat quality, with outcomes dependent on enrichment type and stability. Hanging toys and rotational enrichment consistently improved growth performance, feed efficiency, carcass characteristics, and meat quality parameters, effects likely mediated through increased activity and reduced physiological stress, as evidenced by favorable H/L ratios and metabolic profiles. However, welfare outcomes related to foot condition showed more complex responses. While enrichment did not affect footpad dermatitis or hock burn, toe damage varied markedly among treatments. Stable enrichment (green balls) was associated with lower toe damage scores, whereas frequently altered enrichment (rotational) was linked to increased toe damage, suggesting that increased activity may elevate the risk of localized injuries. These findings indicate that enrichment strategies should be selected based on specific production and welfare objectives. Hanging toys are particularly effective for enhancing growth performance, while simpler, stable enrichment designs may be preferable when minimizing foot injuries is a priority. Future research should investigate interactions among enrichment design, litter quality, and stocking density to optimize both productivity and welfare outcomes.

## Figures and Tables

**Table 1:** Experimental layout of environmental enrichment treatments.

Treatment ID	Treatment Description
T1	Hanging green balls provided from day 1 to day 21
T2	Hanging toy objects provided from day 1 to day 21
T3	Hanging strings provided from day 1 to day 21
T4	Sequential composite enrichment strategy: green balls (days 1–7), hanging toys (days 8–14), and hanging strings (days 15–21)
T5	Control with no environmental enrichment provided

The experiment involved 120,000 birds distributed among five treatments, each consisting of four replicates with 6000 birds per replicate. Treatment 4 was conceived as a single management strategy comprising sequential enrichment phases, and the objective was to evaluate its overall effect across the 21 days rather than to estimate the separate contribution of each object within the rotation.

**Table 2:** Behavioral ethogram used for broiler observations.

Behavior	Description
Active	Bird is moving within the replicate, primarily walking
Inactive	Bird is inactive, standing or sitting without locomotion
Eating	Bird’s head positioned over the feeder
Drinking	Bird’s beak in contact with the drinker
Maintenance	Bird engaged in self-maintenance behaviors, including preening, dust bathing, wing stretching, or scratching

**Table 3:** Effects of different environmental enrichment tools on growth performance of commercial broiler chickens.

Traits	T1	T2	T3	T4	T5	*F*-Value	*p*-Value
IBW, g	40.1 ± 0.20	39.8 ± 0.31	40.6 ± 0.19	40.2 ± 0.24	40.0 ± 0.11	1.97	0.175
1–21 d							
FI, g	1173.3 ± 5.25	1177.9 ± 5.29	1162.7 ± 5.15	1167.9 ± 6.84	1179.3 ± 4.6	1.61	0.247
BW, g	753.9 ^b^ ± 1.40	812.2 ^a^ ± 3.20	752.0 ^b^ ± 1.40	806.6 ^a^ ± 1.10	690.7 ^c^ ± 1.30	711.43	<0.0001
WG, g	713.8 ^c^ ± 1.26	772.4 ^a^ ± 3.21	711.3 ^c^ ± 1.43	766.4 ^b^ ± 1.19	650.7 ^d^ ± 1.38	708.98	<0.0001
FCR	1.64 ^a^ ± 0.011	1.53 ^c^ ± 0.012	1.63 ^b^ ± 0.012	1.52 ^c^ ± 0.013	1.81 ^a^ ± 0.012	194.87	<0.0001
EPEF	131.0 ^b^ ± 0.41	152.2 ^a^ ± 0.90	131.5 ^b^ ± 1.09	151.3 ^a^ ± 0.83	108.9 ^c^ ± 0.84	445.67	<0.0001
22–35 d							
FI, g	1856.5 ± 2.85	1857.3 ± 3.98	1861.0 ± 4.77	1851.9 ± 1.67	1855.8 ± 5.66	0.64	0.645
BW, g	1851.5 ^c^ ± 4.50	1933.9 ^a^ ± 0.80	1832.5 ^d^ ± 2.00	1895.0 ^b^ ± 2.60	1750.8 ^e^ ± 1.30	717.82	<0.0001
WG, g	1097.6 ^b^ ± 3.47	1121.8 ^a^ ± 3.84	1080.6 ^c^ ± 3.00	1088.4 ^c^ ± 1.61	1060.1 ^d^ ± 0.82	65.67	<0.0001
FCR	1.69 ^c^ ± 0.012	1.66 ^d^ ± 0.013	1.72 ^b^ ± 0.013	1.70 ^b,c^ ± 0.014	1.75 ^a^ ± 0.014	23.85	<0.0001
EPEF	312.8 ^c^ ± 2.11	333.7 ^a^ ± 1.97	304.0 ^d^ ± 1.93	318.2 ^b^ ± 0.79	285.7 ^e^ ± 1.18	111.72	<0.0001
1–35 d							
FI, g	3029.9 ± 3.71	3035.2 ± 8.73	3023.7 ± 2.48	3019.8 ± 5.46	3035.1 ± 10.18	1.03	0.437
WG, g	1811.4 ^c^ ± 4.38	1894.1 ^a^ ± 0.63	1791.9 ^d^ ± 1.89	1854.8 ^b^ ± 2.73	1710.8 ^e^ ± 1.38	739.25	<0.0001
FCR	1.67 ^c^ ± 0.015	1.60 ^e^ ± 0.012	1.69 ^b^ ± 0.014	1.63 ^d^ ± 0.015	1.77 ^a^ ± 0.013	205.05	<0.0001
EPEF	316.3 ^c^ ± 1.25	344.8 ^a^ ± 1.18	310.3 ^d^ ± 0.82	332.6 ^b^ ± 1.00	282.0 ^e^ ± 1.38	439.55	<0.0001

Data are presented as least squares means ± standard error of the mean (SEM). Means within a row bearing different superscripts differ significantly at *p* ≤ 0.05. Treatments were as follows: T1, hanging green balls for 21 d; T2, hanging toys for 21 d; T3, hanging strings for 21 d; T4, rotational enrichment (green balls on days 1–7, toys on days 8–14, and strings on days 15–21); and T5, control with no environmental enrichment. IBW = initial body weight; FI = feed intake; WG = weight gain; FCR = feed conversion ratio; EPEF = European production efficiency factor.

**Table 4:** Effects of different environmental enrichment tools on behavioral assessment of commercial broiler chickens.

Traits	T1	T2	T3	T4	T5	*F*-Value	*p*-Value
1–21 d							
Active	24.1 ^a,b^ ± 0.47	23.8 ^a,b^ ± 0.53	22.7 ^b^ ± 0.53	24.7 ^a^ ± 0.49	6.9 ^c^ ± 0.37	252.40	<0.0001
Inactive	26.4 ^b^ ± 0.32	26.2 ^b^ ± 0.34	26.0 ^b^ ± 0.35	25.9 ^b^ ± 0.29	41.1 ^a^ ± 0.5	330.02	<0.0001
Eating	20.2 ^b^ ± 0.46	20.4 ^b^ ± 0.54	21.1 ^b^ ± 0.44	21.1 ^b^ ± 0.48	25.0 ^a^ ± 0.44	17.05	<0.0001
Drinking	19.5 ^b^ ± 0.51	19.6 ^b^ ± 0.53	19.5 ^b^ ± 0.51	20.2 ^b^ ± 0.48	21.8 ^a^ ± 0.30	4.15	0.003
Maintenance	9.9 ^a,b^ ± 0.79	10.0 ^ab^ ± 0.80	10.7 ^a^ ± 0.86	8.1 ^b^ ± 0.78	5.3 ^c^ ± 0.46	8.31	<0.0001
22–35 d							
Active	23.8 ^a^ ± 0.39	23.3 ^a^ ± 0.45	23.7 ^a^ ± 0.46	23.6 ^a^ ± 0.41	6.4 ^b^ ± 0.38	333.31	<0.0001
Inactive	21.8 ^b^ ± 0.44	21.5 ^b^ ± 0.43	22.3 ^b^ ± 0.46	22.2 ^b^ ± 0.43	42.2 ^a^ ± 0.51	398.94	<0.0001
Eating	20.8 ^b,c^ ± 0.35	21.4 ^b,c^ ± 0.39	20.6 ^c^ ± 0.36	21.9 ^a,b^ ± 0.33	22.5 ^a^ ± 0.42	4.39	0.002
Drinking	19.4 ^b^ ± 0.37	19.4 ^b^ ± 0.41	19.9 ^b^ ± 0.34	19.3 ^b^ ± 0.42	23.8 ^a^ ± 0.38	25.54	<0.0001
Maintenance	14.1 ^a^ ± 0.73	14.5 ^a^ ± 0.88	13.5 ^a^ ± 0.75	13.0 ^a^ ± 0.66	5.05 ^b^ ± 0.593	29.40	<0.0001
1–35 d							
Active	24.0 ^a,b^ ± 0.32	23.6 ^a,b^ ± 0.38	23.2 ^b^ ± 0.35	24.2 ^a^ ± 0.31	6.66 ^c^ ± 0.244	558.88	<0.0001
Inactive	24.1 ^b^ ± 0.28	23.8 ^b^ ± 0.28	24.2 ^b^ ± 0.32	24.0 ^b^ ± 0.26	41.7 ^a^ ± 0.36	678.59	<0.0001
Eating	20.5 ^c^ ± 0.29	20.9 ^b,c^ ± 0.32	20.8 ^b,c^ ± 0.28	21.5 ^b^ ± 0.28	23.7 ^a^ ± 0.32	18.87	<0.0001
Drinking	19.5 ^b^ ± 0.29	19.5 ^b^ ± 0.33	19.7 ^b^ ± 0.28	19.7 ^b^ ± 0.32	22.8 ^a^ ± 0.24	23.98	<0.0001
Maintenance	12.0 ^a,b^ ± 0.50	12.2 ^a^ ± 0.57	12.1 ^a^ ± 0.61	10.6 ^b^ ± 0.50	5.19 ^c^ ± 0.373	33.68	<0.0001

Data are presented as least squares means ± standard error of the mean (SEM). Means within a row bearing different superscripts differ significantly at *p* ≤ 0.05. Treatments were as follows: T1, hanging green balls for 21 d; T2, hanging toys for 21 d; T3, hanging strings for 21 d; T4, rotational enrichment (green balls on days 1–7, toys on days 8–14, and strings on days 15–21); and T5, control with no environmental enrichment. Minor deviations from 100% in the reported values reflect statistical averaging of pen-level percentages prior to calculation of treatment means.

**Table 5:** Effects of different environmental enrichment tools on foot condition scores of commercial broiler chickens at 35 days of age.

Traits	T1	T2	T3	T4	T5	χ^2^	*p*-Value
Toe damage	1.64 ^c^ ± 0.062	1.82 ^a,b^ ± 0.056	1.75 ^a,b,c^ ± 0.054	1.89 ^a^ ± 0.046	1.69 ^b,c^ ± 0.057	15.36	0.004
Foot Pad Dermatitis	1.72 ± 0.053	1.78 ± 0.052	1.74 ± 0.058	1.88 ± 0.046	1.67 ± 0.064	9.39	0.051
Hock burn	1.76 ± 0.053	1.79 ± 0.057	1.76 ± 0.055	1.90 ± 0.044	1.76 ± 0.054	6.44	0.168

Data are presented as least squares means ± standard error of the mean (SEM). Means within a row bearing different superscripts differ significantly at *p* ≤ 0.05. Treatments were as follows: T1, hanging green balls for 21 d; T2, hanging toys for 21 d; T3, hanging strings for 21 d; T4, rotational enrichment (green balls on days 1–7, toys on days 8–14, and strings on days 15–21); and T5, control with no environmental enrichment. Each parameter was assessed using a binomial scale ranging from 1 (present) to 2 (not present).

**Table 6:** Effects of different environmental enrichment tools on the blood biochemical profile of commercial broiler chickens at 35 days of age.

Traits	T1	T2	T3	T4	T5	*F*-Value	*p*-Value
Heterophils count	18.7 ^b^ ± 1.64	19.7 ^b^ ± 1.03	18.5 ^b^ ± 1.82	19.8 ^b^ ± 1.7	47.2 ^a^ ± 1.24	68.80	<0.0001
Lymphocytes count	76.2 ^b^ ± 2.50	74.4 ^b,c^ ± 1.34	73.2 ^b,c^ ± 1.38	70.1 ^c^ ± 1.87	86.8 ^a^ ± 1.32	13.24	<0.0001
H/L Ratio	0.25 ^b^ ± 0.022	0.27 ^b^ ± 0.024	0.25 ^b^ ± 0.032	0.28 ^b^ ± 0.021	0.55 ^a^ ± 0.025	36.77	<0.0001
Glucose, mg/dL	184.3 ^b^ ± 0.72	185.4 ^b^ ± 0.68	183.9 ^b^ ± 0.67	185.3 ^b^ ± 0.89	204.6 ^a^ ± 0.61	152.44	<0.0001
Cholesterol, mg/dL	111.3 ^b^ ± 0.93	110.3 ^b^ ± 0.88	109.7 ^b^ ± 0.69	109.3 ^b^ ± 1.48	123.7 ^a^ ± 1.08	34.40	<0.0001
Total Protein, g/dL	4.68 ^b^ ± 0.133	4.68 ^b^ ± 0.091	4.73 ^b^ ± 0.147	4.73 ^b^ ± 0.105	5.95 ^a^ ± 0.063	27.11	<0.0001
Albumin, g/dL	2.30 ^b^ ± 0.115	2.36 ^b^ ± 0.083	2.35 ^b^ ± 0.116	2.37 ^b^ ± 0.073	3.14 ^a^ ± 0.031	17.42	<0.0001
Globulin, g/dL	2.38 ^b^ ± 0.074	2.32 ^b^ ± 0.053	2.37 ^b^ ± 0.056	2.36 ^b^ ± 0.058	2.81 ^a^ ± 0.044	17.05	<0.0001

Data are presented as least squares means ± standard error of the mean (SEM). Means within a row bearing different superscripts differ significantly at *p* ≤ 0.05. Treatments were as follows: T1, hanging green balls for 21 d; T2, hanging toys for 21 d; T3, hanging strings for 21 d; T4, rotational enrichment (green balls on days 1–7, toys on days 8–14, and strings on days 15–21); and T5, control with no environmental enrichment.

**Table 7:** Effects of different environmental enrichment tools on carcass traits of commercial broiler chickens at 35 days of age.

Traits	T1	T2	T3	T4	T5	*F*-Value	*p*-Value
Pre-slaughter weight, g	1853.7 ^a^ ± 5.37	1934.2 ^b^ ± 1.14	1833.4 ^c^ ± 2.42	1896.2 ^d^ ± 2.47	1750.8 ^e^ ± 1.01	558.60	<0.0001
Dressed weight, g	1224.2 ^a^ ± 26.6	1240.0 ^a^ ± 7.67	1208.7 ^a^ ± 21.29	1268.2 ^a^ ± 22.08	1090.1 ^b^ ± 9.87	13.02	<0.0001
Carcass yield, %	66.0 ^a^ ± 1.24	64.1 ^a,b^ ± 0.41	65.9 ^a^ ± 1.11	66.9 ^a^ ± 1.17	62.3 ^b^ ± 0.60	3.67	0.028
Liver weight, g	43.3 ^a^ ± 1.53	46.2 ^a^ ± 1.39	38.9 ^b^ ± 1.51	43.7 ^a^ ± 0.64	35.0 ^c^ ± 0.27	13.89	<0.0001
Heart weight, g	7.9 ^a^ ± 0.11	8.7 ^a^ ± 0.16	7.9 ^a^ ± 0.34	8.0 ^a^ ± 0.42	6.8 ^b^ ± 0.08	7.11	0.002
Gizzard weight, g	26.0 ± 2.15	23.4 ± 2.92	21.1 ± 1.48	23.6 ± 2.73	21.1 ± 0.35	0.93	0.471

Data are presented as least squares means ± standard error of the mean (SEM). Means within a row bearing different superscripts differ significantly at *p* ≤ 0.05. Treatments were as follows: T1, hanging green balls for 21 d; T2, hanging toys for 21 d; T3, hanging strings for 21 d; T4, rotational enrichment (green balls on days 1–7, toys on days 8–14, and strings on days 15–21); and T5, control with no environmental enrichment.

**Table 8:** Effects of different environmental enrichment tools on meat quality traits of commercial broiler chickens at 35 days of age.

Traits	T1	T2	T3	T4	T5	*F*-Value	*p*-Value
Lightness (L*)	54.7 ^a^ ± 1.35	53.7 ^a^ ± 0.40	55.5 ^a^ ± 1.17	54.7 ^a^ ± 0.51	50.8 ^b^ ± 0.31	4.59	0.013
Redness (a*)	13.4 ± 0.39	14.3 ± 0.36	13.1 ± 1.36	14.3 ± 0.47	13.4 ± 0.15	0.64	0.643
Yellowness (b*)	17.7 ± 0.79	18.5 ± 0.63	16.0 ± 0.82	17.0 ± 0.52	16.9 ± 1.20	1.26	0.327
Initial pH	6.41 ^a^ ± 0.014	6.43 ^a^ ± 0.021	6.41 ^a^ ± 0.020	6.41 ^a^ ± 0.014	6.25 ^b^ ± 0.017	23.46	<0.0001
Ultimate pH	5.56 ^c^ ± 0.032	5.63 ^b^ ± 0.019	5.60 ^b,c^ ± 0.024	5.59 ^b,c^ ± 0.017	5.72 ^a^ ± 0.018	11.30	<0.0001

Data are presented as least squares means ± standard error of the mean (SEM). Means within a row bearing different superscripts differ significantly at *p* ≤ 0.05. Treatments were as follows: T1, hanging green balls for 21 d; T2, hanging toys for 21 d; T3, hanging strings for 21 d; T4, rotational enrichment (green balls on days 1–7, toys on days 8–14, and strings on days 15–21); and T5, control with no environmental enrichment.

## Data Availability

The original contributions presented in this study are included in the article. Further inquiries can be directed to the corresponding author(s).
